# ss-siRNAs allele selectively inhibit ataxin-3 expression: multiple mechanisms for an alternative gene silencing strategy

**DOI:** 10.1093/nar/gkt693

**Published:** 2013-08-09

**Authors:** Jing Liu, Dongbo Yu, Yuichiro Aiba, Hannah Pendergraff, Eric E. Swayze, Walt F. Lima, Jiaxin Hu, Thazha P. Prakash, David R. Corey

**Affiliations:** ^1^Departments of Pharmacology and Biochemistry, UT Southwestern Medical Center at Dallas, Dallas, TX 75390, USA and ^2^Department of Medicinal Chemistry and Core Antisense Research, ISIS Pharmaceuticals, Carlsbad, CA 92010, USA

## Abstract

Single-stranded silencing RNAs (ss-siRNAs) provide an alternative approach to gene silencing. ss-siRNAs combine the simplicity and favorable biodistribution of antisense oligonucleotides with robust silencing through RNA interference (RNAi). Previous studies reported potent and allele-selective inhibition of human huntingtin expression by ss-siRNAs that target the expanded CAG repeats within the mutant allele. Mutant ataxin-3, the genetic cause of Machado–Joseph Disease, also contains an expanded CAG repeat. We demonstrate here that ss-siRNAs are allele-selective inhibitors of ataxin-3 expression and then redesign ss-siRNAs to optimize their selectivity. We find that both RNAi-related and non-RNAi-related mechanisms affect gene expression by either blocking translation or affecting alternative splicing. These results have four broad implications: (i) ss-siRNAs will not always behave similarly to analogous RNA duplexes; (ii) the sequences surrounding CAG repeats affect allele-selectivity of anti-CAG oligonucleotides; (iii) ss-siRNAs can function through multiple mechanisms and; and (iv) it is possible to use chemical modification to optimize ss-siRNA properties and improve their potential for drug discovery.

## INTRODUCTION

Synthetic nucleic acids drugs have long been an attractive concept for drug development (1), which have the potential to bind specific sequences within RNA and regulate expression of almost any gene. Such regulation might have a major impact on therapeutics, but major clinical successes have been elusive, and excitement has been often matched by skepticism. In January 2013, the Food and Drug Administration (FDA) approved Kynamro, a synthetic antisense oligonucleotide (ASO) to treat familiar hypercholesterolemia (2). Kynamro is systemically administered in saline without the need for formulation. Its therapeutic profile demonstrates that synthetic nucleic acids can inhibit expression of disease genes in patients and reduce target protein levels sufficiently to affect the course of the disease.

Like any pharmaceutical candidate, oligonucleotides require optimization to achieve the potencies and selectivities needed to unlock many applications. Existing approaches for gene silencing include duplex RNAs and ASOs (1). Duplex RNAs (dsRNAs) function through the RNA interference (RNAi) pathway and are robust tools for controlling gene expression in cell culture. In animals, good effects can be achieved when duplex RNAs are used in complex with nanoparticles (3). RNA-nanoparticle formations are advancing in clinical trials, but the need for multiple components may slow progress and widespread adoption. In the absence of nanoparticle complexes, duplex RNA activity in animals requires concentrations that will usually be too high to consider during human therapy.

ASOs like Kynamro are also achieving success in clinical trials (1,2). A strength of ASOs is that no formulation is necessary and they can be administered in saline. For silencing RNAs (siRNAs), an advantage is that there is a dedicated cellular machinery to efficiently recognize their targets, and it is reasonable to hypothesize that function through the RNAi machinery will sometimes have the potential to deliver better drugs. A challenge has been to develop compounds that combine the robust silencing of siRNA with the simplicity and favorable biodistribution of ASOs.

In 2002, Zamore (4) and Tuschl (5) reported that unmodified single-stranded RNA could function inside cells to inhibit gene expression. In these examples, potency was much lower than with analogous duplex RNAs, probably because of the inherent instability of single-stranded RNA when exposed to extracellular and intracellular enzymes. Subsequent studies showed that chemically modified single-stranded RNA could also achieve gene silencing (6–10). Potencies, however, remained low, and there were few follow-up studies to examine their mechanism or generality.

**Figure 1.**
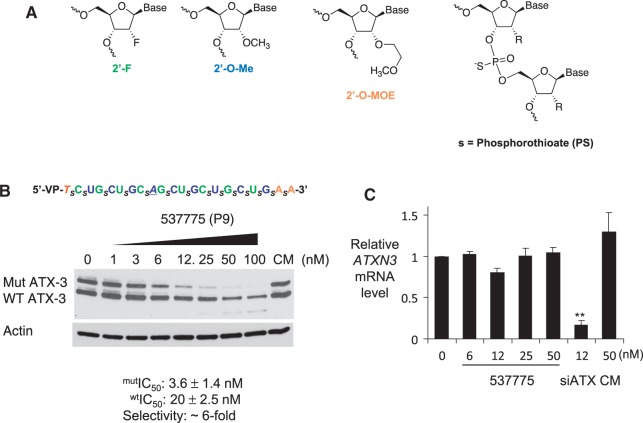
A benchmark ss-siRNA is an allele-selective inhibitor of ATX-3 expression in GM06151 patient-derived fibroblasts. (**A**) Structures of chemically modified bases and PS linkages in ss-siRNA. Underlined bases are mismatched relative to the CAG repeat. Subscript ‘_s_’ indicates PS linkage; Green, 2′-Fluoro; Blue, 2′-O-methyl; Orange, 2′-O-methoxyethyl. All other sugars are ribose and all other linkages are phosphate. (**B**) Sequence and inhibitory effect of ss-siRNA ISIS 537775 on protein or (**C**) RNA expression. Error bars on ATX-3 mRNA levels are standard deviations (SD) from independent replicate data. Western analysis data are representative of triplicate experiments. CM: non-complementary duplex RNA. siATX: positive control duplex RNA that is complementary to a sequence with ATX3 mRNA outside of the trinucleotide repeat. Statistic significance was calculated by *t*-test. **P < 0.01 relative to negative control CM.

In 2012, Lima and colleagues (11) discovered a pattern of phosphorothioate (PS) (Figure 1A), 2′-fluoro (2′-F), and 2′-O-methyl (2′-O-Me) modifications that yielded RNA single-strands capable of entering the protein machinery of the RNA-induced silencing complex and inhibiting gene expression with potencies approaching those of RNA duplexes. They termed these compounds single-stranded siRNAs (ss-siRNAs). Introduction of a metabolically stable 5′-(E)-vinylphosphonate moiety to mimic a natural 5′ phosphate allowed efficient gene silencing inside animals. This study showed that iterative design optimization could achieve dramatic improvements in the properties of single-stranded RNA.

Our laboratory used ss-siRNAs to efficiently silence expression of huntingtin (HTT) protein (12). HTT causes Huntington’s disease (HD), an incurable neurological disorder (13). The mutated allele contains an expanded CAG repeat within the protein-encoding region of HTT mRNA. Our ss-siRNA was complementary to the CAG repeat region. We showed that the anti-CAG ss-siRNA recruited argonaute 2 (AGO2) protein to *HTT* mRNA and caused selective inhibition of mutant HTT in patient-derived human fibroblast cells and in HD mouse model (12). The most potent ss-siRNAs had centrally located mismatches relative to the CAG repeat. These mismatches were designed to make the ss-siRNAs function more like endogenous miRNAs by preventing cleavage of target mRNA (14). In a second study, we showed that ss-siRNAs could also act in cell nuclei to target non-coding transcripts at the progesterone receptor promoter and inhibit progesterone receptor gene expression (15).

HD is one of several diseases caused by expansion of a CAG repeat (13,16). Another is spinocerebellar ataxia-3 (SCA3) (also known as Machado-Joseph Disease). SCA3 is a progressive neurodegenerative disorder caused by an expanded repeat within one allele of the gene encoding ataxin-3 (ATX-3) protein (17), and allele-selective inhibition can be achieved using duplex RNAs that target polymorphisms (18,19). We had previously shown that mismatch-containing duplex RNAs targeted to the CAG repeat were potent and allele-selective inhibitors of ATX-3 expression (20).

We now test anti-CAG ss-siRNAs for inhibition of ATX-3 expression. One goal for these studies was to examine the hypothesis that a single anti-CAG ss-siRNA can be a lead compound for developing therapies for multiple trinucleotide expansion diseases by expanding inhibition to ATX-3. A second goal was to understand similarities and differences between duplex RNAs and ss-siRNAs during allele-selective inhibition of gene expression. We find that ss-siRNAs can be allele-selective inhibitors of ATX-3. Surprisingly, however, several ss-siRNAs also function through a second mechanism that alters splicing of ATX-3. Our data suggest that, depending on their design and exact pattern of chemical substitutions, ss-siRNAs can function through a mixture of silencing mechanisms, and experimental outcomes can be tailored by strategic choice of ss-siRNA design.

## MATERIALS AND METHODS

### Synthesis of ss-siRNAs

ssRNA syntheses were performed on ABI 394 synthesizer (1–2 µmol scale) by the phosphoramidite coupling method on an UnyLinker solid support packed in the column. A 0.1 M solution of 2′-F, 2′-*O*-Me and 2′-*O*-MOE nucleoside phosphoramidites in anhydrous CH_3_CN were used for the synthesis. For the coupling step, the phosphoramidites were delivered 6–9-fold excess over the loading on the solid support, and phosphoramidite condensation was carried out for 10 min. All other steps in the protocol supplied by manufacturer were used. A solution of 3% dichloroacetic acid in dichloromethane was used for removing dimethoxytrityl group from 5′-hydroxyl group of the nucleotide. Extended detritylation condition was used to remove the dimethoxytrityl group from the secondary hydroxyl group of the UnyLinker solid support. The 4,5-Dicyanoimidazole (0.7 M) in anhydrous CH_3_CN was used as activator during coupling step. PS linkages were introduced using 0.2 M solution of phenylacetyl disulfide in 1:1 pyridine/CH_3_CN as sulfur transfer reagent and treated for 3 min. A solution of *tert*-butyl hydroperoxide/acetonitrile/water (10:87:3) was used to introduce phosphodiester linkages and treated for 12 min. Chemical phosphorylation reagent procured form Glen Research Inc., Virginia, USA was used to phosphorylate the 5′-terminus of ss-siRNAs. The step-wise coupling efficiencies were >97%.

After completion of the synthesis, solid support was suspended in aqueous ammonia (28–30 wt. %) and heated at 55°C for 6 h. The reaction mixture was allowed to come to room temperature, and the solid support was filtered and washed with water. The washing and filtrate were combined together and evaporated to dryness. The residue obtained was dissolved in water and purified by High Performance Liquid Chromatography (HPLC) on a strong anion exchange column (Mono Q, GE Healthcare, 16/10, 20 ml, 10 µm, ionic capacity 0.27–0.37 mmol/ml, A = 100 mM ammonium acetate, 30% aqueous acetonitrile, B = 1.5 M NaBr in A, 0–60% B in 40 min, Flow 1.5 ml min^−^^1^, λ = 260 nm). Desalting by HPLC on a reverse phase column gave ss-siRNAs in an isolated yield of 15–30% based on the initial loading on the solid support. ss-siRNAs were characterized by ion-pair-HPLC coupled mass spectrometry (MS) analysis with Agilent 1100 MSD system.

### Cell culture and transfection

ss-siRNAs (11,12) and bridged nucleic acids (BNAs) were synthesized by ISIS Pharmaceuticals (Carlsbad, CA) and reconstituted in nuclease-free water. Patient-derived fibroblast cell lines GM06151 were obtained from the Coriell Institute (Camden, NJ). The fibroblasts were maintained at 37°C and 5% CO_2_ in Minimal Essential Media Eagle (Sigma, M4655) supplemented with 10% heat inactivated fetal bovine serum (Sigma) and 0.5% non-essential amino acids (Sigma). Cells were plated at a density of 70 000 per well of a 6-well plate 48 h before transfection. siRNAs were transfected into cells with lipid RNAiMAX (Invitrogen) as previously described (20). Cells were typically harvested 3 days after transfection for qPCR or 4 days for protein assay. For double transfection experiments, the first transfection was performed as described. Media was changed 24 h later, and cells were split into new 6-well plate after 72 h of transfection. The second transfection was carried out on the next day. Media was changed again after 24 h, and cells were harvested after 96 h of second transfection for protein analysis.

### Western blot and PCR analysis

In all, 7.5% or 4-20% acrylamide pre-cast gels (Bio-Rad) were used to separate the ATX-3 isoforms. The primary antibodies were used: anti-ATX-3 (MAB5360, Millipore), anti-ATX-3 polyclonal antibody (a gift from Dr Henry Paulson, University of Michigan), anti-polyQ monoclonal antibody (5TF1-1C2, Millipore, MAB1574) and anti-β-actin (Sigma). Protein bands were quantified using ImageJ software. The percentage of inhibition was calculated as a relative value to a control sample. Dose fitting curve was generated using GraphPad Prism 4 program by the equation: y = 100[1-x^m^/(n^m ^+ x^m^)], where y is percentage of inhibition, and x is the siRNA concentration, n is the IC_50_ value, and m is the Hill coefficient value. All the experiments were repeated for at least three times, and the error bar is standard deviation.

Quantitative PCR was performed on a 7500 real-time PCR system (Applied Biosystems) using iTaq SYBR Green Supermix (Bio-rad). Data were normalized relative to levels of Glyceraldehyde 3-phosphate dehydrogenase (GAPDH) mRNA. The following qPCR primers were used: 5′–GGAAATATGGATGACAGTGG–3′(F); 5′–ATCCTGAGCCTCTGATACTC–3′(R). GAPDH primers were obtained from Applied Biosystems. The qPCR cycles are as follows: 50°C for 2 min; 95°C for 5 min; (95°C for 15s; 60°C for 1 min) × 40 cycles. Experiments were performed in biological triplicate and error reported as standard deviation. For RT-PCR, the amplification was performed using LA Taq DNA polymerase (TaKaRa) using the following primers to detect the spliced band of ATX-3: P8 primer pair 5′-GATGAGGAAGCAGATCTCCGCAGGG-3′(8F), 5′-CTAAAGACATGGTCACAGCTGCCTGAAGC-3′(8R); P10 primer pair 5′-GATTTGCAGAGGGCTCTGGCACTAAGTC-3′(10F) and 5′-AGCATGTCTTCTTCACTCATAGCATCACTTTTC-3′(10R). The PCR products were separated on 1.5% agarose gels and visualized on an AlphaImager.

### RNA immunoprecipitation

SCA3 fibroblast cells were seeded at 1400 K in 150 cm^2^ dishes. Duplex RNAs were transfected with RNAiMAX in the next day. Cells were harvested 72 h later and were lysed in a buffer [20 mM Tris–HCl (pH7.4) 150 mM NaCl, 2 mM MgCl_2_, 0.5% NP-40, 0.5 mM DTT, protease inhibitor (EDTA-free, Roche) and RNase inhibitor (Promega, 50 U/ml final)] with a volume about three times of the cell pellet size. The mixture was sat on ice for 10 min after thorough mixing. After centrifugation, the supernatant were isolated and stored at −80°C. Sixty microliters of Protein A/G agarose Plus was incubated with 4 µl of antibodies (anti-AGO2, 4G8, 011-22033, Wako; anti-GW-182, A302-329A, Bethyl Laboratories; or mouse IgG, 12-371, Millipore) in 1 × PBS (pH 7.4) at 4°C with gentle agitation for 2 h. After two washes of 1 × PBS, beads were incubated with cell lysate for 2 h at 4°C. The beads were extensively washed with aforementioned lysis buffer once, IP wash buffer twice [300 mM NaCl, 3 mM MgCl_2_, 0.5% NP-40 and 20 mM Tris–HCl (pH 7.4) and 1 × PBS once. The beads were finally eluted with elution buffer (1% SDS, 0.1 M NaHCO_3_ and RNase inhibitor]. After proteinase K treatment, RNA extraction and precipitation, samples were treated with recombinant DNase I, followed by reverse transcription. The mRNA levels were quantified by qPCR. Results were normalized first by GAPDH levels and second by that of IgG.

## RESULTS

### Allele-selective inhibition of ATX-3 by an ss-siRNA

We monitored ATX-3 expression in SCA3 patient-derived fibroblast cell line GM06151. GM06151 cells are heterozygous for the mutant ATX-3 allele, with 24 CAG repeats on the wild-type allele and 74 repeats on the mutant allele. Seventy-four repeats is near the mean repeat number found in patients with SCA3 (17). ss-siRNAs or double stranded RNA (dsRNA) were transfected into adherent cells using cationic lipid and were harvested 4 days after transfection for visualization by western blot and quantitation.

ss-siRNAs are heavily chemically modified with a mixture of 2′-F, 2′-*O*-Me and 2′-*O*-methoxyethyl (2′-MOE) substitutions (Figure 1a) (11,22). The 2′-O-Me nucleotides can be substituted for 2′-MOE if necessary. They also contain a mixture of phosphodiester and PS internucleotide linkages. These modifications patterns were designed to combine improved metabolic stability inside cells with ability to successfully interact with cellular RNAi machinery. A phosphate mimic, 5′-(*E*)-vinylphosponate, is necessary for activity in animals but is not necessary for activity in cell culture. Many of the ss-siRNAs used in these studies contained a 5′-phosphate because the synthesis of phosphate modified ss-siRNAs is more straightforward and facilitates testing a substantial numbers of compounds.

We had previously observed that duplex RNAs that were fully complementary to the CAG repeat showed little or no selectivity for inhibiting the mutant *HTT* allele (23,24). As a result, we altered bases within the duplex to be mismatched relative to the CAG target, reasoning that this would switch gene silencing to the mechanism used by miRNAs and possibly allow greater allele selectivity. Instead of the slicer mechanism used by AGO2, when RNAs were fully complementary to their targets (25,26), the imperfect base-pairing permits binding by AGO2 but not cleavage of the target mRNA. These introduced mismatches led to improved selectivity for duplex RNAs and ss-siRNAs (12,15,24,27,28).

We first examined a benchmark ss-siRNA that had been extensively evaluated in our previous work for inhibition of HTT (12). ISIS 537775 is an ss-siRNA with a 5′-(*E*)-vinylphosphonate and a single mismatch at position 9. For inhibition of HTT expression, ISIS 537775 possessed a selectivity of >29-fold and an IC_50_ value of 3.5 nM. For ATX-3, inhibition was characterized by a selectivity of 6-fold and an IC_50_ value of 3.6 nM (Figure 1B, Supplementary Figure S1). These data suggest ss-siRNAs can selectively inhibit the expression of mutant ATX-3, but that selectivity may be lower than that previously observed for HTT. Consistent with disruption of the slicer activity of AGO2 by introduction of a central mismatch (14), levels of ATX-3 mRNA were unchanged over a range of sss-siRNA concentrations (Figure 1C).

### Some ss-siRNAs produce a higher mobility product

**Figure 2.**
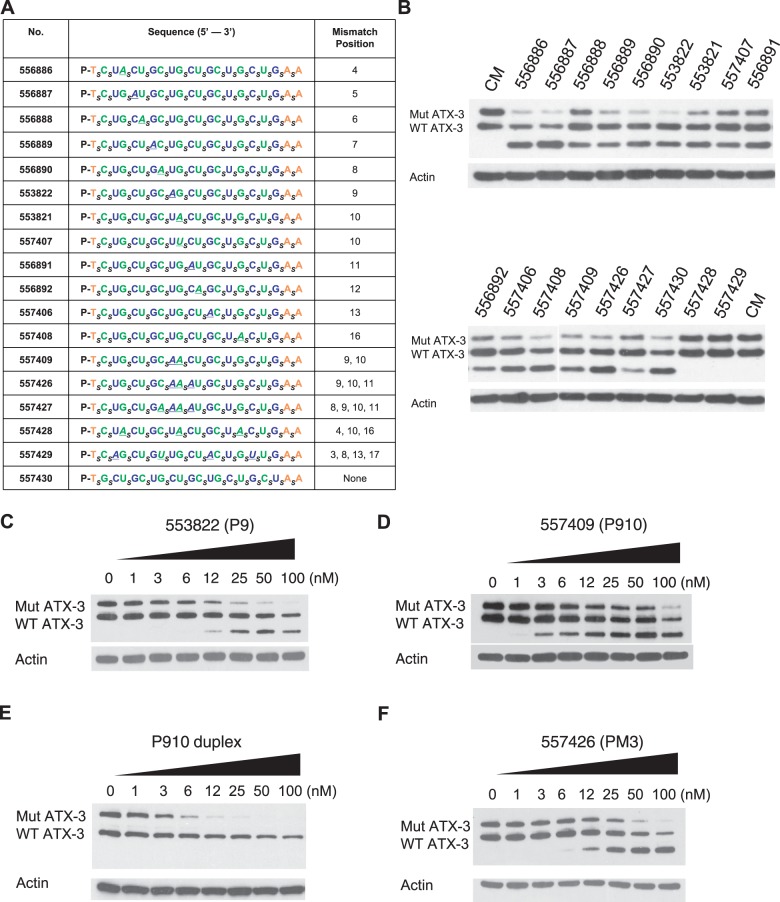
Inhibition of ATX-3 expression in GM06151 patient-derived fibroblasts by ss-siRNAs with one or more mismatched bases. (**A**) List of ss-siRNAs. Subscript ‘_s_’ indicates PS linkage; Green, 2′-F; Blue, 2′-O-Me; Orange, 2′-MOE. The terminal T has a 5′ phosphate. All other sugars are ribose and all other linkages are phosphate. (**B**) Effects of ss-siRNAs tested at 25 nM. Effect on ATX-3 expression of increasing concentrations of (**C**) ISIS 553822, (**D**) ISIS 557409, (**E**) a duplex RNA with no chemically modified bases or PS linkages analogous to ISIS 557409 and (**F**) ISIS 557426. Western analysis data (C–F) is representative of triplicate experiments that were averaged to yield IC_50_ and selectivity values. CM: non-complementary duplex RNA.

We screened additional ss-siRNAs to help understand how placement of mismatches affected potency and allele selectivity. Single mismatches were systematically placed throughout the ss-siRNAs or introduced in groups of two or three (Figure 2A). All compounds contained a 5′-phosphate rather than a 5′-(*E*)-vinyl phosphonate to simplify the synthesis of multiple compounds. Previously, we had tested these ss-siRNAs for inhibiting expression of HTT and identified many compounds with allele selectivities from 5 to greater than 30-fold selectivity (12).

We screened the analogs at 25 nM to identify the most promising compounds for further analysis (Figure 2B). ISIS 553822 was a 5′-phosphate analog of ISIS 537775 and possessed similar IC_50_ and allele selectivity values of 8.4 nM and 12-fold, respectively (Figure 2C, Supplementary Figure S2). By contrast, other compounds that had been selective when used to inhibit expression of HTT, or that had duplex RNA analogs that were selective for inhibition of mutant ATX-3 expression, showed little allele selectivity for ATX-3.

For example, ISIS 557409 possessed only a 4-fold selectivity for inhibition of mutant *ATX-3* expression (Figure 2D). By contrast to 4-fold selectivity for mutant ATX-3, the selectivity of ISIS 557409 for mutant HTT was >16-fold (12). In addition, the 4-fold selectivity for ss-siRNA ISIS 557409 for ATX-3 is in contrast to 16-fold selectivity achieved by the analogous duplex RNA (Figure 2E). ISIS 557426, a compound that possesses three central mismatches, also showed little selectivity for mutant ATX-3 (Figure 2F), even though it showed >30-fold selectivity for HTT (12), and the analogous duplex RNA was 10-fold selective for ATX-3 (20).

Surprisingly, for ISIS 553822, ISIS 557409 and ISIS 557426, we observed formation of a higher mobility band. This band is also visible, although less obviously, on treatment with the more selective compound ISIS 537775 (Figure 1B). We had not observed a higher mobility band during our previous experiments with 16 different anti-CAG duplex RNAs (Figure 2E shows an example of a duplex analogous to ISIS 557409) (20), suggesting that mechanisms of gene silencing by duplex RNA and ss-siRNA may be significantly different.

### Design optimization to improve potency and selectivity

Successful preclinical testing of compounds that inhibit ATX-3 expression would benefit from the identification of compounds that have optimal potencies and selectivities. Our initial screening suggested that achieving high selectivity for inhibition of ATX-3 would be more difficult than for inhibition of HTT. Several strategies are available for optimizing anti-CAG ss-siRNAs. These include varying length, changing the pattern/number/type of chemical modifications, and altering the placement of mismatched bases.

**Figure 3.**
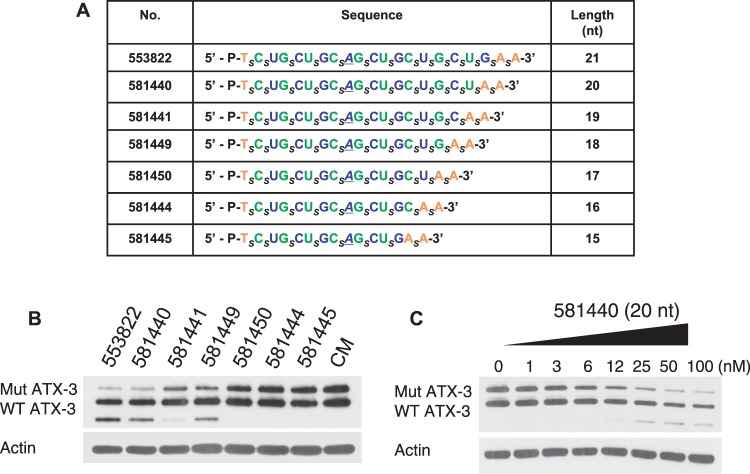
Inhibition of ATX-3 expression in GM06151 patient-derived fibroblasts by ss-siRNAs of varied length. (**A**) List of ss-siRNAs. Subscript ‘_s_’ indicates PS linkage; Green, 2′-F; Blue, 2′-O-Me; Orange, 2′-MOE. The terminal T has a 5′ phosphate. All other sugars are ribose, and all other linkages are phosphate. (**B**) Effect of ss-siRNAs tested at 25 nM, representative data from triplicate determinations. (**C**) Effect of increasing concentrations of ISIS 581440. CM: non-complementary duplex RNA.

We tested ss-siRNAs that were based on 21 base ISIS 553822 but were 15–20 bases in length (Figure 3A). As ss-siRNA length was trimmed from 21 to 15 nucleosides, T_m_ values varied dramatically, decreasing from 85°C for ISIS 553822 to 67/68°C for ISIS 581445. Of the six compounds tested (Figure 3A and B), only 20 base ISIS 581440 possessed a potency (12 nM) and allele selectivity (6-fold) similar to ISIS 533822 (Figure 3C, Supplementary Figure S3). The loss of potency as length is reduced is consistent with the reduced binding affinity of the shorter ss-siRNAs and their lesser potential to interact efficiently with either the mRNA target or the RNAi machinery.

**Figure 4.**
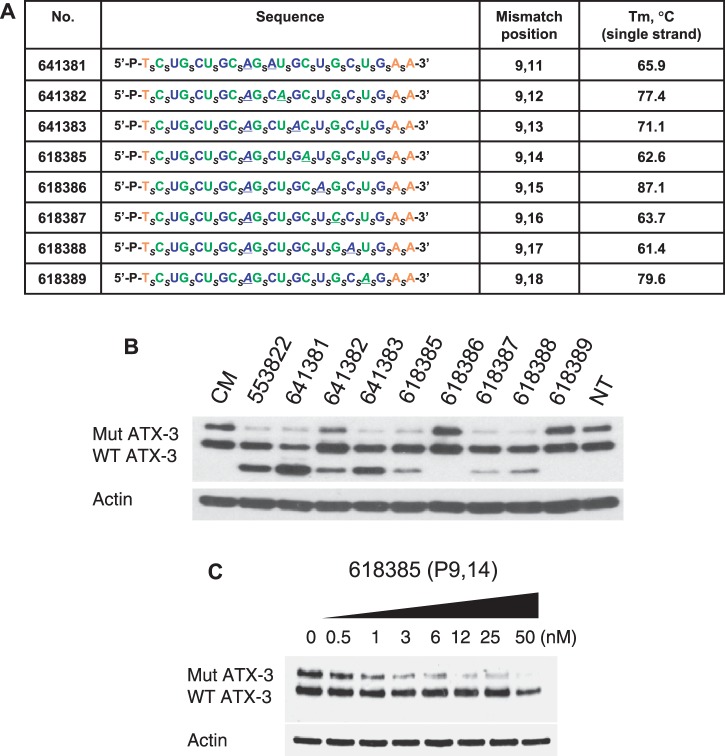
Inhibition of ATX-3 expression in GM06151 patient-derived fibroblasts by ss-siRNAs with systematically introduced mismatched bases. (**A**) List of ss-siRNAs. Subscript ‘_s_’ indicates PS linkage; Green, 2′-F; Blue, 2′-O-ME; Orange, 2′-MOE. The terminal T has a 5′ phosphate. All other sugars are ribose, and all other linkages are phosphate. (**B**) Effects of ss-siRNAs tested at 25 nM. (**C**) Effect of increasing concentration of ISIS 618385, representative data from triplicate experiments. CM: non-complementary duplex RNA. NT: No treatment.

We then designed a series of compounds that combined a mismatch at position 9 with mismatches at other positions (Figure 4A). Our goal was to test the hypothesis that subtle changes in ss-siRNA:mRNA complementarity would impact allele selectivity. We found that, even though the doubly substituted ss-siRNAs were based on the same parent compound, they differed in allele selectivity and propensity to produce a higher mobility band (Figure 4B, Supplementary Figure S4). The most allele-selective compounds, ISIS 618385, showed excellent potency and possessed an allele selectivity of >35-fold (Figure 4C). This allele selectivity is one of the highest we have noted for any compound for any CAG-repeat target gene, demonstrating that ss-siRNA design can be optimized to produce substantially improved agents.

### Improving potency by relieving self-structure

It was striking that two compounds that combined a second mismatch with a P9 mismatch, ISIS 618386 and ISIS 618389, showed little inhibition of either the mutant or wild-type allele. Inspection of both ss-siRNAs revealed the potential to form a hairpin or self-pairing duplex with unusually high energy, suggesting that a reduced ability to adopt an unbasepaired conformation might be preventing efficient recognition.

**Figure 5.**
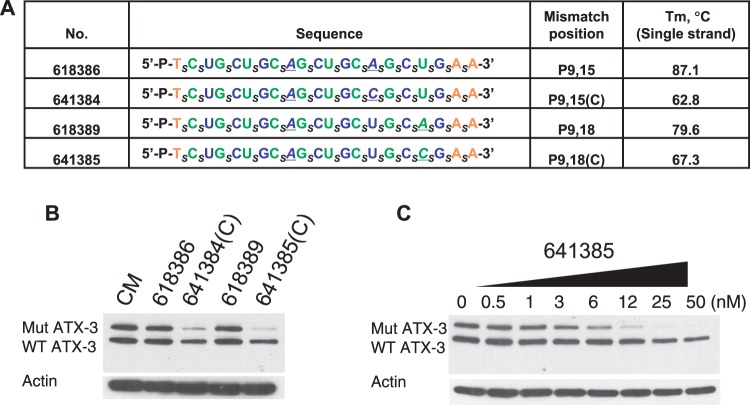
Effect of modifying ss-siRNAs to alter secondary structure and affect allele selectivity in GM06151 patient-derived fibroblasts. (**A**) List of ss-siRNAs. Subscript ‘_s_’ indicates PS linkage; Green, 2′-F; Blue, 2′-O-Me; Orange, 2′-MOE; Black, unmodified nucleoside. The terminal T has a 5′ phosphate. All other sugars are ribose and all other linkages are phosphate. (**B**) Effects of ss-siRNAs tested at 25 nM. (**C**) Effect of increasing concentrations of ISIS 641385, representative data from triplicate determinations. CM: non-complementary duplex RNA.

To test this hypothesis, we introduced a single A to C substitution in both ss-siRNAs to create new compounds ISIS 641384 and ISIS 641385 (Figure 5A) and test the impact of hairpin formation. As predicted, these changes lowered the measured T_m_ values for the single strands annealing to themselves. In contrast to the lack of activity of the parent compounds, but consistent with their lower potential for intramolecular structure, both derivatives were effective silencing agents when tested at 25 nM (Figure 5B). ISIS 641384 was only 3-fold allele selective, but ISIS 641385 had a 2.1 nM potency and >23-fold selectivity and showed no formation of the higher mobility band (Figure 5C, Supplementary Figure S5). These data further demonstrate that the position and the identity of the base substitution can be rationally manipulated to improve allele-selective inhibition.

### Effect of altering chemical modifications

**Figure 6.**
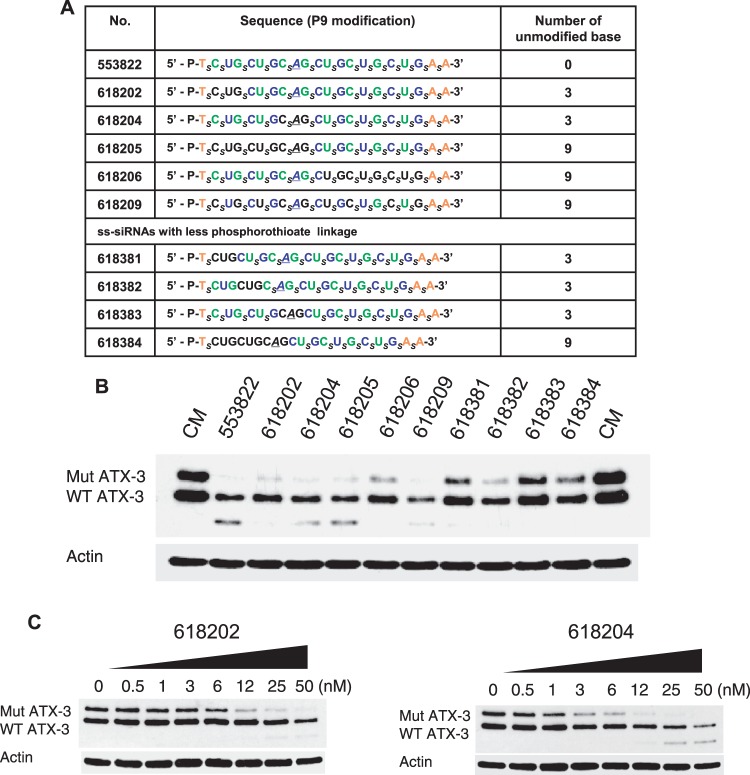
Effect of changing chemical modification of ss-siRNAs on allele-selective inhibition of ATX-3 in GM06151 patient-derived fibroblasts. (**A**) Table of ss-siRNAs. Subscript ‘_s_’ indicates PS linkage; Green, 2′-F; Blue, 2′-O-Me; Orange, 2′-MOE; Black, unmodified nucleoside. The terminal T has a 5′ phosphate. All other sugars are ribose, and all other linkages are phosphate. (**B**) Effects of ss-siRNAs tested at 25 nM. Effect of increasing concentrations of (**C**) ISIS 618202 and ISIS 618204, representative data from triplicate determinations. CM: non-complementary duplex RNA.

Another potential strategy for ss-siRNAs is to alter the number and type of chemically modified bases. We tested the effect of increasing number of unmodified RNA bases from 0 to 9 and increasing the number of phosphodiester linkages from 3 to 9 (Figure 6A). All compounds were based on parent compound ISIS 553822 with a mismatch at position 9. Several of these compounds showed selectivities that were similar to ISIS 553822 (Figure 6B, Supplementary Figure S6), demonstrating that the design of active ss-siRNAs is compatible with diverse combinations of natural and modified nucleotides and internucleotide linkages. For example, ISIS 618202 and ISIS 618204 that possessed three unmodified RNA bases had a selectivities of 6- and >16-fold, respectively (Figure 6C). In parallel with improved selectivity, formation of the higher mobility band was reduced for almost every design, suggesting that relatively subtle changes in the chemistry of an ss-siRNA affect the mechanism of gene silencing.

### Involvement of RNAi factors

**Figure 7.**
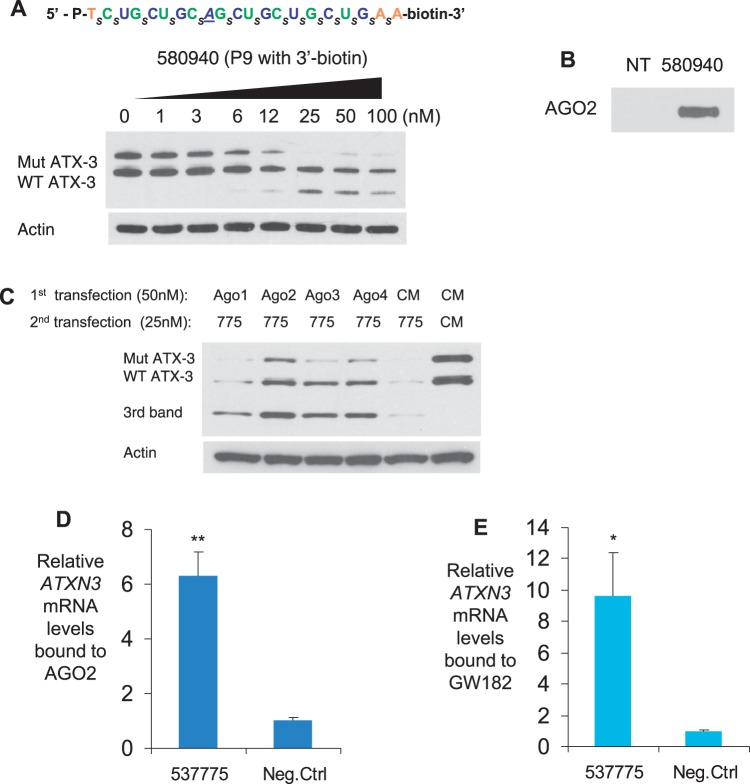
Interactions of ss-siRNAs with RNAi factors AGO2 and GW182 in GM06151 patient-derived fibroblasts. (**A**) Biotin-labeled ss-siRNA ISIS 580940 inhibits ATX-3 expression. (**B**) Western blot image showing pull-down of AGO2 after incubation with biotinylated ISIS 580940 and a biotin:strepavidin purification. (**C**) Effect of reducing AGO1∼4 expression to the function of ss-siRNA 537775 on ATX-3 expression. Anti-AGO siRNAs and negative control siRNA CM were transfected first in SCA3 patient fibroblasts. After 3 days, ss-siRNA 537775 was added. (**D** and **E**). RIP assay examining the involvement of AGO2 or GW182 in ss-siRNA directed mRNA targeting. SCA3 patient fibroblast cells (GM06151) were treated with 25 nM of ss-siRNAs 537775 and a negative control. RNA was isolated from immunoprecipitation of anti-AGO2 or anti-GW182 antibodies. The levels of AGO2 or GW182-associated *ATXN3* mRNA were quantified by qPCR. CM: non-complementary duplex RNA. Statistic significance was calculated by *t*-test. **P < 0.01, *P < 0.05 relative to negative control. For (D and E) the negative control was an ss-siRNA with a sequence unrelated to ATX-3.

To examine the mechanism of ss-siRNAs, we examined the identity of proteins that bind to ss-siRNAs. To accomplish this, we synthesized ISIS 580940, a 3′-biotin-labeled version of ISIS 553822 containing a mismatched base at position 9. On transfection into patient-derived fibroblast cells, ISIS 580940 inhibited expression of mutant ATX-3 with an IC_50_ value of 5.9 nM and an allele selectivity of 7-fold (Figure 7A, Supplementary Figure S7), values similar to analogous non-biotinylated ss-siRNA ISIS 537775. After cell lysis, biotinylated ss-siRNA was recovered using a streptavidin column, and bound proteins were analyzed by gel electrophoresis. Western analysis using anti-AGO2 antibody confirmed the presence of AGO2 in material recovered after streptavidin-biotin purification (Figure 7B).

There are four AGO variants in human cells, AGO1-4. To test involvement of AGO2 and investigate involvement of AGO1, AGO3 and AGO4, we used siRNAs designed to reduce expression of each AGO variant. We found that reduced levels of cellular AGO2, and to a lesser extent AGO3 and AGO4, led to reduced inhibition by ss-siRNA (Figure 7C). Concurrently, levels of the lower molecular weight third band increased. The finding that decreased AGO2 expression increases formation of the lower molecular weight band suggests that an additional silencing mechanism may also be contributing, as decreasing AGO2 levels would be expected to reduce product formation if formation depended on RNAi.

To further confirm involvement of AGO2, we used RNA immunoprecipitation (RIP) with an anti-AGO2 antibody. We observed that addition of ss-siRNA 537775 to cells promoted recruitment of AGO2 to *ATX-3* mRNA (Figure 7D). GW182 (TNRC6A) is another RNAi factor that has been shown to bind AGO protein and be essential for translational silencing by miRNAs (29). There are three GW182 paralogs in human cells, TNRC6A, TNRC6B and TNRC6C. We have previously observed that GW182 is essential for allele-selective silencing of mutant HTT expression by duplex RNA (27). RIP using an anti-GW182 antibody showed that addition of ss-siRNA 537775 caused recruitment of GW182 to *ATX-3* mRNA (Figure 7E).

### The lower molecular weight product is an abbreviated ATX-3 lacking the poly glutamine tract encoded by the CAG repeat

The presence of a lower molecular weight product is an important variable associated with inhibition of ATX-3 by ss-siRNAs. Production of the third band may have an impact on therapeutic applications and is also likely to offer significant insights into mechanism. ss-siRNAs are designed to act like duplex RNAs and function through the RNAi pathway. However, ss-siRNAs are single stranded and contain many of the same chemical modifications that are used by ASOs. It is possible that more than one inhibitory mechanism might be operating.

ASOs fall into two general mechanistic classes (1). One class has a central region of DNA bases capable of forming a DNA–RNA hybrid with mRNA, causing recruitment of RNAse H and cleavage of the target transcript. The other class has chemically modified sugars that prevent cleavage by RNase H distributed throughout the ASO.

This latter class of ASO’s does not cause mRNA degradation and can affect gene expression through two different mechanisms. In one mechanism, the ASO binds mRNA, blocks the ribosome and halts translation. In a second mechanism, the ASO can bind pre-mRNA, block association of splicing factors and alter splicing. ‘Steric blocking’ ASOs do not cause mRNA degradation.

**Figure 8.**
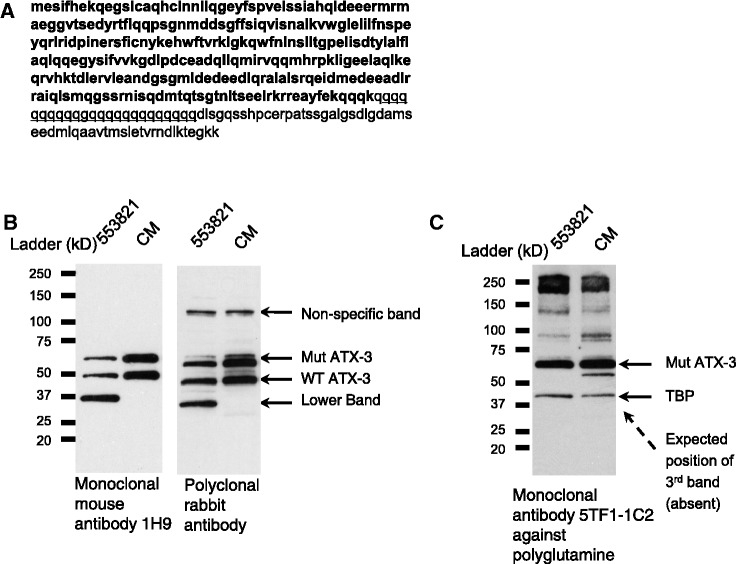
Identity of the lower molecular weight band produced by some anti-CAG ss-siRNAs. (**A**) Amino acid sequence of ATX-3 protein, with boldface highlighting those before the polyglutamine tract (underlined). (**B**, **C**) Western blot images of ss-siRNA treated samples using anti-ATX-3 or anti-polyQ antibodies. The lower molecular weight band migrate at ∼35 kDa. Experiments used in GM06151 patient-derived fibroblasts. CM: non-complementary duplex RNA. 553821: CAG-targeting ss-siRNA with mismatch at position 10.

To test whether steric interference was a plausible explanation for the lower molecular weight band, we examined its size and compared it with the size of potential fragments of ATX3 (Figure 8A). Using two different anti-ATX-3 antibodies for detection, we observed that the lower molecular weight band migrates at ∼35 kDa, a molecular weight that is similar to the predicted ∼34 kDa molecular weight of fragments truncated at the polyglutamine tract encoded by the CAG repeat (Figure 8B). The band was seen regardless of whether a monoclonal or a polyclonal anti-ATX-3 antibody was used, strongly suggesting that the band is a shorter fragment of ATX-3. Antibody 5TF1-1C2 that specifically detects regions of expanded polyglutamines did not detect the lower molecular weight band (Figure 8C), indicating that the product does not include the expanded repeat. These observations are consistent with the third band being either a truncated product that terminates at the CAG repeat or an alternatively spliced variant that excludes the CAG repeat.

### ss-siRNAs can induce alternative splicing

**Figure 9.**
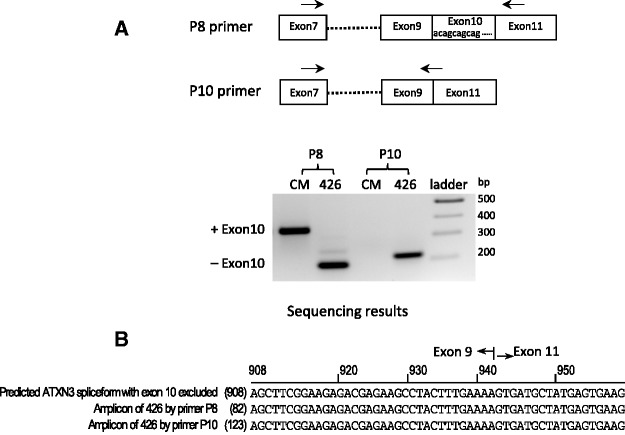
ss-siRNA induces *ATXN3* exon10 exclusion in GM06151 patient-derived fibroblasts. (**A**) *top,* Location of, the CAG repeat within exon 10 and the target sites for the P8 and P10 primer sets; *bottom*, PCR image using primer sets P8 or P10 after treating with ss-siRNA ISIS 557426 (426) and negative control CM. (**B**). Sequencing results of the PCR amplicon of primers P8 and P10.

The CAG region of ATX-3 is located at near the 5′ end of exon 10 within *ATX-3* mRNA. Many ASOs have been identified that alter splicing by recognizing sequences near splice junctions (30). We hypothesized that ss-siRNAs might be acting like ASOs to affect splicing and that, if this was true, the effect on splicing would be apparent at the level of mature *ATX-3* mRNA. To test this hypothesis, we design primers for PCR that were complementary to either side of the CAG repeat. These primers revealed the formation of alternatively spliced products lacking exon10 (Figure 9A and B). The products were appropriately sized and sequencing confirmed the predicted splice junction.

**Figure 10.**
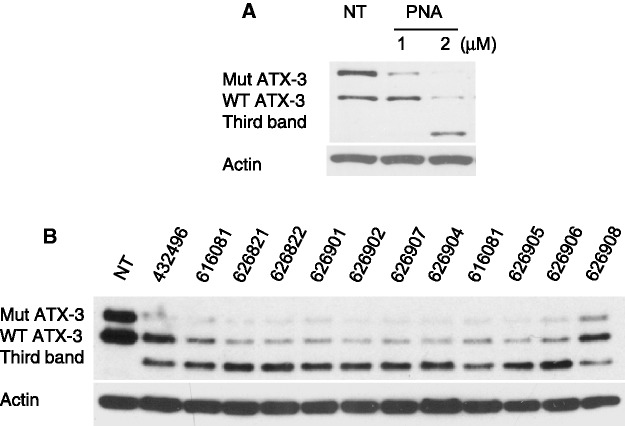
Treatment of GM06151 patient-derived fibroblasts with (**A**) PNAs and (**B**) BNAs also leads to formation of the third band. PNAs were added at 1 or 2 µM. BNAs were added at 25 nM. NT: no treatment.

Peptide nucleic acid (PNA) and oligonucleotides containing BNA nucleosides are also known to affect splicing. In this study, the BNA oligomers contained (S)-cEthyl nucleosides (31). We transfected PNA and BNA oligomers (Supplementary Figure S8) into cells to see whether these oligomers, which cannot function through the RNAi pathway, cause formation of the third band. For both an anti-CAG PNA (Figure 10A) and several different anti-CAG BNAs (Figure 10B), we observed formation of the third band. This is consistent with the hypothesis that the third band is formed by a steric blocking mechanism rather than through delivery by RNAi factors.

## DISCUSSION

### ss-siRNAs, an alternate approach to gene silencing

ss-siRNAs combine favorable properties of ASOs and duplex RNAs. Like ASOs, ss-siRNAs are single stranded, simplifying their synthesis and possibly leading to more favorable biodistribution. Like duplex RNAs, ss-siRNAs function through the RNAi pathway, possibly leading to more robust and potent silencing and increasing the options for obtaining high levels of allele selectivity. These advantages were displayed in our initial report on allele-selective inhibition of human HTT. To date, however, there have been only three studies using ss-siRNAs (11,12,32). Little is known about how their single-stranded nature might cause their mechanism of action to differ from that of analogous duplex RNAs.

### Allele-selective inhibition of ATX-3: a case study for optimizing ss-siRNAs

Mutant HTT and ATX-3 both contain expanded CAG repeats. In our previous work, we had used duplex RNAs to achieve allele-selective inhibition of HTT or ATX-3 protein expression (20,24,27) or ss-siRNAs (12) to inhibit HTT expression. Here, we focused on the development of ss-siRNAs for allele-selective inhibition of ATX-3.

We had previously shown that mismatch-containing duplex RNAs could inhibit mutant ATX-3 expression with allele selectivities as high as 16-fold (20). These allele selectivities were less than those achieved for the inhibition of HTT expression by the same duplexes (24). The RNA sequences surrounding the CAG repeat differ between *HTT* and *ATX-3* mRNAs, and the lower allele selectivities hinted that it might be more difficult to achieve high allele selectivity for blocking ATX-3 expression.

The experiments that we report here support that suggestion. For inhibition of HTT expression, many different ss-siRNAs with single mismatches achieved allele selectivities of 6–30-fold (12). For ATX-3, most single mismatch ss-siRNAs showed little or no allele selectivity, and the best selectivity was just 6-fold, even though HTT and ATX3 mRNA both contain similar CAG repeats. Although many ss-siRNAs achieved greater than 20-fold selectivity for mutant HTT, only two ss-siRNAs showed greater than 20-fold selectivity for ATX-3. However, one of these two ss-siRNAs was >35-fold allele selective for ATX-3, equal to the highest selectivities obtained for mutant HTT and showing that design and testing can lead to outstanding inhibitors even for relatively difficult targets

These data offers further evidence that the feasibility of targeting a CAG repeat is influenced by the sequences that surround it. ss-siRNAs were also less allele selective than corresponding duplex RNAs, even though both can recognize targets through the RNAi pathway. This outcome suggests that the modified nucleotides that allow an ss-siRNA to function inside cells can affect the course of gene silencing and lead to subtle and not so subtle differences relative to analogous duplex RNAs.

The lower allele selectivity for inhibition of mutant ATX-3 of the ss-siRNAs tested in our first studies is a challenge to therapeutic development and led us to investigate whether it would be possible to improve selectivity. Fortunately, oligonucleotide synthesis is versatile. Many different derivatives can be synthesized with different chemical modifications or altered location of mismatched bases. Both strategies produced more allele-selective ss-siRNAs, with allele selectivities as high as for any compounds, regardless of gene target or silencing mechanism. Although identification of highly allele-selective ss-siRNAs was not as routine for inhibition of ATX-3 as it had been for HTT, the identification of inhibitors with selectivities of greater than 20–30-fold was readily accomplished.

Currently, there are no crystal structures for ss-siRNA bound to AGO2. Examination of crystal structures of AGO2 bound to RNA did not suggest to us where chemical modifications or mismatches might be introduced to improve selectivity. Rational choice of changes was further handicapped by the observation that selectivity differed depending on the substrate, with *ATX-3* mRNA being more challenging than *HTT* mRNA.

In the absence of adequate structural insights, the best approach seems to be the synthesis and testing of systematically modified compounds and development of structure activity relationships that indicate the types of changes that have the potential to yield compounds with improved selectivity and facilitates iterative cycles of redesign and compound improvement. This approach is commonly used in small molecule drug design and refining the modification pattern of ss-siRNAs is likely to yield even better compounds. The synthesis of ss-siRNAs is relatively straightforward and obtaining the number and variety of compounds necessary to identify improved agents was not unusually difficult.

The finding that subtle changes in chemistry and substitution pattern can improve allele selectivity supports the conclusion that ss-siRNAs have substantial flexibility to be tailored for individual applications to maximize potency and selectivity. Although beyond the scope of this study, it seems reasonable to conclude that a variety of chemically modified ss-siRNA designs are compatible with efficient gene silencing and that these designs provide many options for improving biodistribution or other *in vivo* properties.

### ss-siRNAs can combine antisense mechanisms

Identification of a higher-mobility ATX-3 product was an unanticipated outcome of this project. We had not previously observed an alternate protein product during our work with duplex RNAs or ss-siRNA inhibitors of HTT expression or duplex RNA inhibitors of ATX-3 expression. The product appears to be a splice variant, and formation of the variant seems to be independent of the ability of the ss-siRNAs to act through the RNAi pathway.

**Figure 11.**
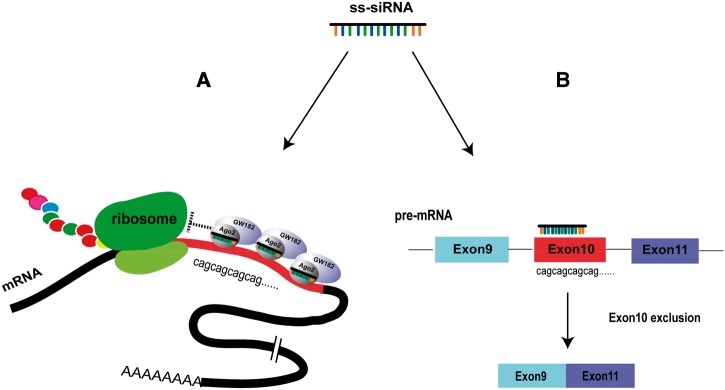
Scheme showing mechanisms of action for ss-siRNAs. (**A**) ss-siRNAs can associate with RNAi factors, bind to CAG repeats and block translation. (**B**) Alternatively ss-siRNAs can bind to CAG repeats independent of RNAi factors and cause formation of an alternatively splicing product.

The ability of single-stranded ASOs to affect splicing is well-known (30). Although ss-siRNAs are designed to be able to function through the RNAi pathway, it is essential to realize that the chemical modifications that are critical for their stability inside cells also allow them to function as ASOs. It is reasonable to assume that there will always be a competition for an ss-siRNA between having recognition facilitated by association with RNAi factors (Figure 11A) and recognition occurring through an antisense mechanism that does not require RNAi factors (Figure 11B). For the latter possibility, ss-siRNAs may bind alone or they may recruit proteins other than RNAi factors (32).

Our data suggest that the RNAi-independent steric-blocking mechanism can prevail for some ss-siRNAs for inhibiting ATX-3 expression. This mechanism reduces allele selectivity and leads to formation of a higher mobility product. Formation of the higher mobility band varies dramatically depending on which ss-siRNA is used, further demonstrating that seemingly subtle modifications to ss-siRNA design can have large implications for function. Experimenters can choose whether the RNAi or steric-antisense mechanisms predominate by modulating ss-siRNA chemistry.

Recent work from van Roon-Mom and co-workers has shown that exon-skipping for *ATX-3* can also be achieved using ASOs with 2′-O-methyl nucleotides and PS internucleotide linkages (33). In this study, an ASO targeting exon 10 outside of the CAG repeat was able to induce skipping of exon 10, consistent with our observation that recognition of exon 10 by ASOs can lead to formation of an alternatively spliced product. This work went on to show that the combination of two ASOs targeting both exons 9 and 10 yielded skipping of both exons and a protein that lacked the CAG repeat but retained normal ubiquitin binding.

### Moving forward with ss-siRNAs

Although the concept of gene silencing by nucleic acids is simple, the development of highly effective compounds and understanding their mechanism is not easy. Like any other type of synthetic compound that is intended to function inside cells, it is important to synthesize and test many different derivatives to identify the best compounds and gain insights into their mechanisms of action. Such systematic testing is especially important for new classes of compounds like ss-siRNAs or anti-CAG oligomers because so little is known about their properties.

We show here that highly selective inhibitors of mutant ATX-3 expression can be identified through the synthesis and testing of many different ss-siRNAs. We show that it is possible to reduce the number of chemically modified nucleotides, and future work may show that other chemical modifications will also be compatible with ss-siRNA function. Although ss-siRNAs function through the RNAi pathway, they can also function through a steric-block antisense mechanism independently of RNAi to produce a shorter ATX-3 variant. This possibility introduces one more variable into the development of ss-siRNAs, but also an opportunity to broaden their ability to control target gene expression.

It is plain that our understanding of the properties of ss-siRNAs is at an early stage. Much more work remains to understand how to achieve optimal results, but our initial studies suggest the many options for design will make ss-siRNAs a rich source for developing agents to silence gene expression.

## SUPPLEMENTARY DATA

Supplementary Data are available at NAR Online.

## FUNDING

Work in the Corey Laboratory was supported by the National Institutes of Health [NIGMS 73042]; an award from the McKnight Foundation for Neuroscience, Cure Huntington's Disease Initiative (CHDI) Inc. Foundation Inc., and the Robert A. Welch Foundation [I-1244]; supported by a Young Investigator Award from the National Ataxia Foundation (J.H.); supported by a Japan Society for the Promotion of Science Postdoctoral Fellowship (to Y.A.). Funding for open access charge: NIH [NIGMS 73042].

*Conflict of interest statement*. None declared.

## Supplementary Material

Supplementary Data
