# Supplementary material for: ss-siRNAs allele selectively inhibit ataxin-3 expression: multiple mechanisms for an alternative gene silencing strategy
Source: Nucleic Acids Res. 2013 Aug 9;41(20):9570–83. doi: 10.1093/nar/gkt693 (PMC3814390; doi:10.1093/nar/gkt693)
Supplement: Supplementary Data [file supp_41_20_9570__index.html]

ss-siRNAs allele selectively inhibit ataxin-3 expression: multiple mechanisms for an alternative gene silencing strategy — ss-siRNAs allele selectively inhibit ataxin-3 expression: multiple mechanisms for an alternative gene silencing strategy — Supplementary Data 

# ss-siRNAs allele selectively inhibit ataxin-3 expression: multiple mechanisms for an alternative gene silencing strategy

## Supplementary Data

files

**Files in this Data Supplement:**

- Supplementary Data - pdf file
