# Supplementary material for: ss-siRNAs allele selectively inhibit ataxin-3 expression: multiple mechanisms for an alternative gene silencing strategy
Source: Nucleic Acids Res. 2013 Aug 9;41(20):9570–83. doi: 10.1093/nar/gkt693 (PMC3814390; doi:10.1093/nar/gkt693)
Supplement: Supplementary Data [file supp_gkt693_nar-01671-f-2013-File002.pdf]

Supplementary Figure 1. Averaged dose-curves of ss-siRNA 537775 on ataxin-3 expression. ss-siRNA was transfected into GM06051 patient derived fibroblast cells at increased concentrations. Data was obtained from at least three independent experiments.

5’-VP-T$_s$C$_s$UG$_s$CU$_s$GC$_s$*A*G$_s$CU$_s$GC$_s$U$_s$G$_s$C$_s$U$_s$G$_s$A$_s$A-3’

537775 (P9)

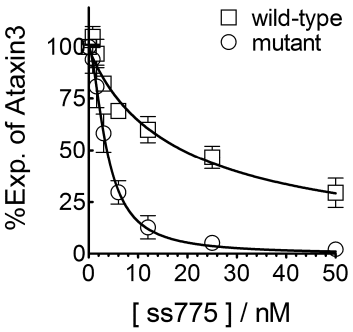

$^{mut}IC_{50}$:3.6 ± 1.4 nM
$^{wt}IC_{50}$:20 ± 2.5 nM
Selectivity: ~ 6-fold

Supplementary Figure 2. Averaged dose-curves of ss-siRNAs 553882, 557409, 557426 and P910 duplex RNA on ataxin-3 expression. siRNAs were transfected into GM06051 patient derived fibroblast cells at increased concentrations. Data was obtained from at least three independent experiments.

A

553822 (P9)

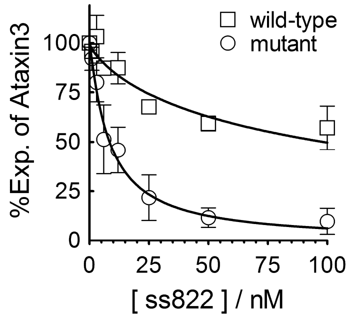

$^{mut}IC_{50}$: 8.4 ± 2.5 nM
$^{wt}IC_{50}$: 99 ± 28 nM
Selectivity: ~12-fold

B

557409 (P910)

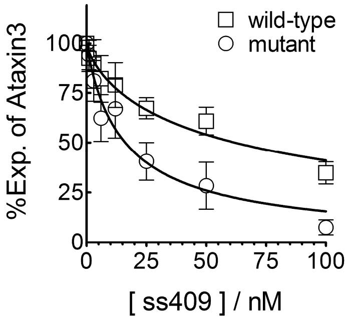

$^{mut}IC_{50}$: 16 ± 3.5 nM
$^{wt}IC_{50}$: 62 ± 16 nM
Selectivity: ~4-fold

C

557426 (PM3)

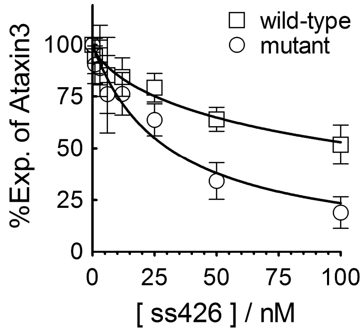

$^{mut}IC_{50}$: 30 ± 6.5 nM
$^{wt}IC_{50}$: > 100 nM
Selectivity: >3-fold

D

P910 duplex

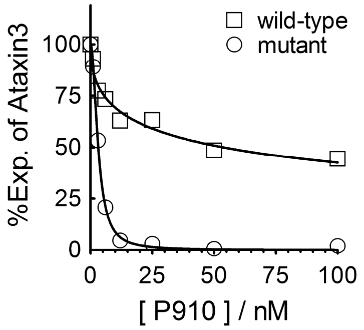

$^{mut}IC_{50}$: ~ 3.3 nM
$^{wt}IC_{50}$: ~ 52.5 nM
Selectivity: ~16-fold

Supplementary Figure 3. Averaged dose-curves of 20mer ss-siRNA 581440 on ataxin-3 expression. ss-siRNA was transfected into GM06051 patient derived fibroblast cells at increased concentrations. Data was obtained from at least three independent experiments.

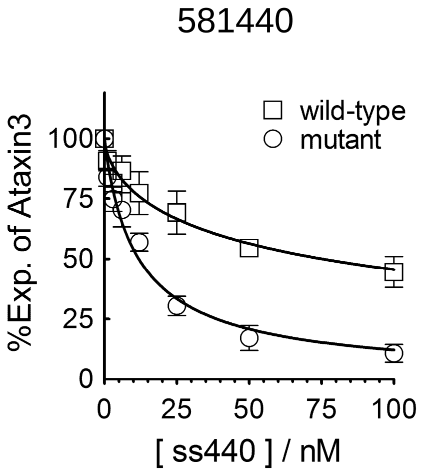

$^{mut}IC_{50}$: 12 ± 1.2 nM
$^{wt}IC_{50}$: 75 ± 18 nM
Selectivity: ~6-fold

Supplementary Figure 4. Averaged dose-curves of ss-siRNA with two separate mismatches 641381 and 618385 on ataxin-3 expression. ss-siRNA was transfected into GM06051 patient derived fibroblast cells at increased concentrations. Data was obtained from at least three independent experiments.

A

641381(P9,11)

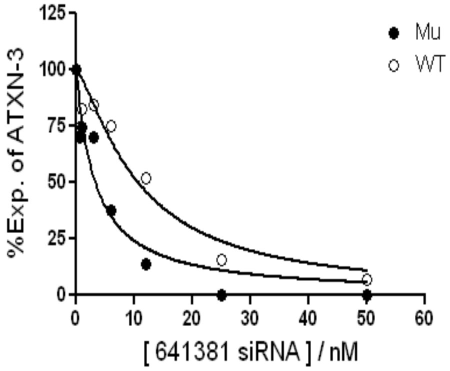

$^{mut}IC_{50}$: 3.3 ± 0.8 nM
$^{wt}IC_{50}$: 10 ± 2.3 nM
Selectivity: ~3-fold

B

618385(P9,14)

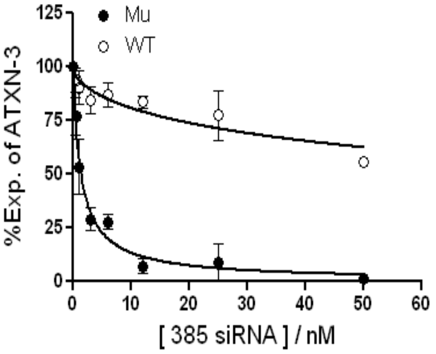

$^{mut}IC_{50}$: 1.4 ± 0.2 nM
$^{wt}IC_{50}$: > 50 nM
Selectivity: >35-fold

Supplementary Figure 5. Effects of modifying ss-siRNAs to alter secondary structure and affect allele-selectivity. Averaged dose-curves of ss-siRNAs 641384 and 641385 on ataxin-3 expression. ss-siRNA was transfected into GM06051 patient derived fibroblast cells at increased concentrations. Data was obtained from at least three independent experiments.

A 641384(P9, 15C)

B 641385(P9,18C)

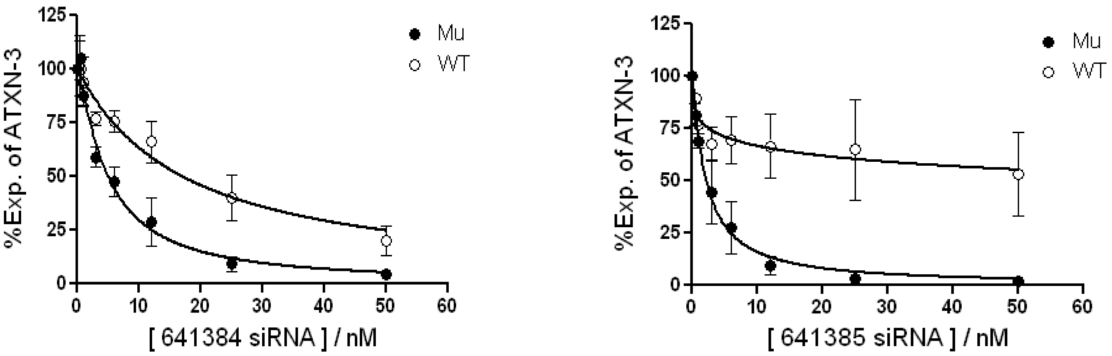

A: $^{mut}IC_{50}$: 5.0 ± 0.6 nM
$^{wt}IC_{50}$: 17 ± 2.8 nM
Selectivity: ~3-fold

B: $^{mut}IC_{50}$: 2.1 ± 0.3 nM
$^{wt}IC_{50}$: > 50 nM
Selectivity: > 23-fold

Supplementary Figure 6. Effects of changing chemical modification of ss-siRNAs on allele-selective inhibition of ATX-3. Averaged dose-curves of ss-siRNAs 618202, 618204, 618205 and 618209 ataxin-3 expression. ss-siRNA was transfected into GM06051 patient derived fibroblast cells at increased concentrations. Data was obtained from at least three independent experiments.

A

618202

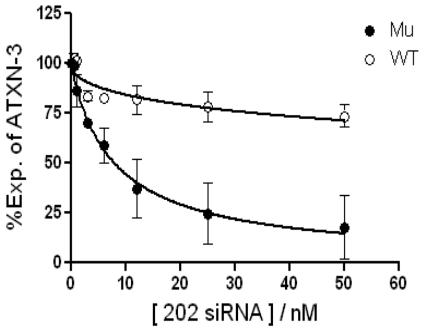

$^{mut}IC_{50}$: 7.9 ± 1.4 nM
$^{wt}IC_{50}$: > 50 nM
Selectivity: >6-fold

B

618204

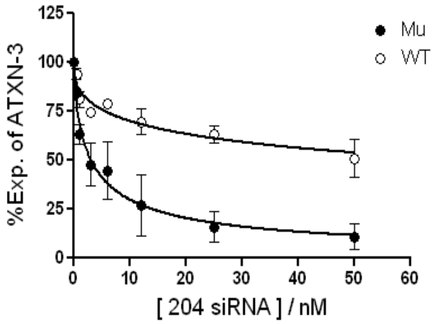

$^{mut}IC_{50}$: 3.1 ± 0.8 nM
$^{wt}IC_{50}$: > 50 nM
Selectivity: >16-fold

C

618205

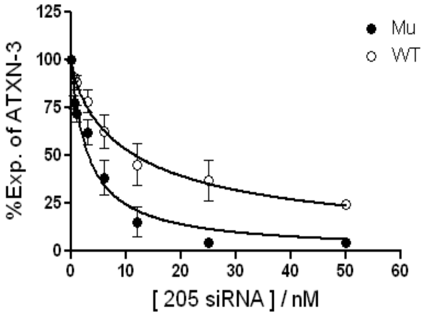

$^{mut}IC_{50}$: 3.1 ± 0.4 nM
$^{wt}IC_{50}$: 11 ± 1.5 nM
Selectivity: ~3-fold

D

618209

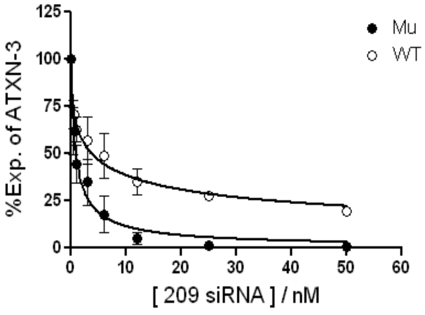

$^{mut}IC_{50}$: 0.9 ± 0.1 nM
$^{wt}IC_{50}$: 3.8 ± 0.8 nM
Selectivity: ~4-fold

E

618382

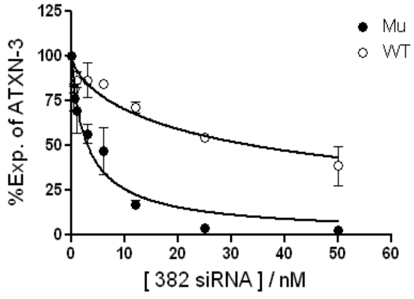

$^{mut}IC_{50}$: 3.1 ± 0.6 nM
$^{wt}IC_{50}$: 33 ± 7.8 nM
Selectivity: ~11-fold

Supplementary Figure 7. Interaction of ss-siRNAs with RNAi factors AGO2 and GW182 in GM06051 patient derived fibroblast cells. Averaged dose-curves of biotinylated ss-siRNA 580940 on ataxin-3 expression. Data was obtained from at least three independent experiments.

580940 (P9 with 3'-biotin)

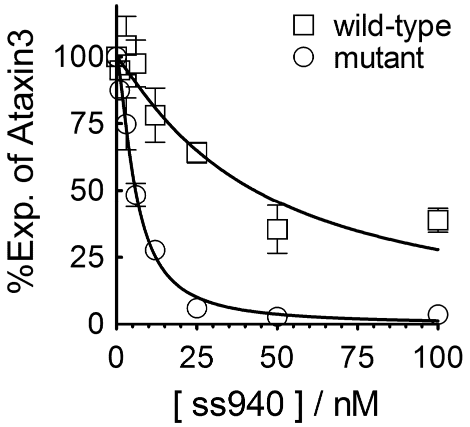

$^{mut}IC_{50}$: 5.9 ± 1.7 nM

$^{wt}IC_{50}$: 43 ± 7.0 nM

Selectivity: ~7-fold

Supplementary Figure 8. Structures and sequences of (A) PNA and (B) cEt BNAs.

A

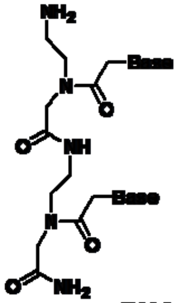

PNA

5'-lys-GCTGCTGCTGCTGCTGCTG-lys$_{8}$-3'

B

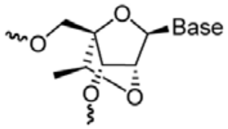

(*S*)-cEt bridged nucleic acid (cEt)

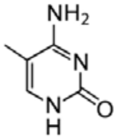

5-methyl cytosine (mC) base

Isis #	Sequence (5'-3')	Chemistry
432496	$G_{ds}C_{ds}U_{ks}G_{ds}C_{ds}U_{ks}G_{ds}C_{ds}U_{ks}G_{ds}C_{ds}U_{ks}G_{ds}C_{ds}U_{ks}G_{ds}C_{ds}U_{ks}G_{d}$	$d_2kd_2kd_2kd_2kd_2kd$
616081	$G_{ds}{}^{m}C_{ds}T_{ks}G_{ds}{}^{m}C_{ds}T_{ks}G_{ds}{}^{m}C_{ds}T_{ks}G_{ds}{}^{m}C_{ds}T_{ks}G_{ds}{}^{m}C_{ds}T_{ks}G_{ds}{}^{m}C_{ds}T_{ks}G_{d}$	$d_2kd_2kd_2kd_2kd_2kd$
626821	$G_{ks}{}^{m}C_{ds}T_{ks}G_{ds}{}^{m}C_{ds}T_{ks}G_{ds}{}^{m}C_{ds}T_{ks}G_{ds}{}^{m}C_{ds}T_{ks}G_{ds}{}^{m}C_{ds}T_{ks}G_{ds}{}^{m}C_{ds}T_{ks}G_{k}$	$kdkd_2kd_2kd_2kd_2kd_2k_2$
626822	$G_{ks}{}^{m}C_{ds}T_{ks}G_{ds}{}^{m}C_{ks}T_{s}G_{ks}{}^{m}C_{ds}T_{ks}G_{ds}{}^{m}C_{ks}T_{s}G_{ks}{}^{m}C_{ds}T_{ks}G_{ds}{}^{m}C_{ks}T_{ds}G_{k}$	kdkdkdkdkdkdkdkdkdk
626901	${}^{m}C_{ks}T_{ds}G_{ks}{}^{m}C_{ds}T_{ds}G_{ks}{}^{m}C_{ds}T_{s}G_{ks}{}^{m}C_{ds}T_{s}G_{ks}{}^{m}C_{ds}T_{ds}G_{ks}{}^{m}C_{ds}T_{ds}G_{ks}{}^{m}C_{k}$	$kdkd_2kd_2kd_2kd_2kd_2k_2$
626902	${}^{m}C_{ks}T_{ds}G_{ks}{}^{m}C_{ds}T_{ks}G_{ds}{}^{m}C_{ks}T_{ds}G_{ks}{}^{m}C_{ds}T_{ks}G_{ds}{}^{m}C_{ks}T_{s}G_{ks}{}^{m}C_{ds}T_{ks}G_{ds}{}^{m}C_{ks}$	kdkdkdkdkdkdkdkdkdk
626907	$T_{ks}G_{ds}{}^{m}C_{ks}T_{s}G_{ks}{}^{m}C_{ds}T_{s}G_{ks}{}^{m}C_{ds}T_{s}G_{ks}{}^{m}C_{ds}T_{s}G_{ks}{}^{m}C_{ds}T_{ds}G_{ds}{}^{m}C_{ks}T_{k}$	$kdkd_2kd_2kd_2kd_2kd_2k_2$
626904	$T_{ks}G_{ds}{}^{m}C_{ks}T_{s}G_{ks}{}^{m}C_{ds}T_{ks}G_{ds}{}^{m}C_{ks}T_{s}G_{ks}{}^{m}C_{ds}T_{ks}G_{ds}{}^{m}C_{ks}T_{ds}G_{ks}G_{ds}{}^{m}C_{k}$	kdkdkdkdkdkdkdkdkdk
BNA with less phosphorothioate linkages		
626905	$G_{ks}{}^{m}C_{ds}T_{ko}G_{ds}{}^{m}C_{ds}T_{ko}G_{ds}{}^{m}C_{ds}T_{ko}G_{ds}{}^{m}C_{ds}T_{ko}G_{ds}{}^{m}C_{ds}T_{ko}G_{ds}{}^{m}C_{ds}T_{ks}G_{k}$	$kdkd_2kd_2kd_2kd_2kd_2k_2$
626906	${}^{m}C_{ks}T_{ds}G_{ko}{}^{m}C_{ds}T_{ds}G_{ko}{}^{m}C_{ds}T_{ds}G_{ko}{}^{m}C_{ds}T_{s}G_{ko}{}^{m}C_{ds}T_{ds}G_{ko}{}^{m}C_{ds}T_{ds}G_{ks}{}^{m}C_{k}$	$kdkd_2kd_2kd_2kd_2kd_2k_2$
626908	$T_{ks}G_{ds}{}^{m}C_{ko}T_{s}G_{ds}{}^{m}C_{ko}T_{s}G_{ds}{}^{m}C_{ko}T_{s}G_{ds}{}^{m}C_{ko}T_{s}G_{ds}{}^{m}C_{ko}T_{ds}G_{ds}{}^{m}C_{ks}T_{k}$	$kdkd_2kd_2kd_2kd_2kd_2k_2$

o= phosphodiester, s = phosphorothioate,
k = cEt BNA, d = DNA, ${}^{m}C$ = 5-methyl cytosine
